# RBM23 Drives Hepatocellular Carcinoma by Activating NF-*κ*B Signaling Pathway

**DOI:** 10.1155/2021/6697476

**Published:** 2021-03-17

**Authors:** Hexu Han, Ting Lin, Ziyi Fang, Guoxiong Zhou

**Affiliations:** ^1^Department of Gastroenterology, Affiliated Hospital of Nantong University, Nantong University, Jiangsu 226001, China; ^2^Department of Pathophysiology, School of Medicine, Nantong University, Jiangsu 226001, China

## Abstract

**Purpose:**

Hepatocellular carcinoma (HCC) is a leading cause of cancer-related death worldwide, and angiogenesis has been proven to be significantly involved in its progression. However, the molecular mechanism underlying HCC angiogenesis has not been well researched. In this study, RNA Binding Motif Protein 23 (RBM23) was identified as a novel proangiogenic factor in HCC cell lines and tissues.

**Materials and Methods:**

Firstly, we analyzed the correlation of clinical specimens. In HCC tissues, the levels of RBM23 and microvessel density (MVD) showed a strong positive correlation. Furthermore, data from related cytology experiments showed that the knockdown of RBM23 expression in HCC cells significantly inhibited the tube formation by the human vascular endothelial cells in vitro. The mechanism of this phenomenon was found to be through increasing the mRNA of p65 and enhanced the nuclear accumulation of p65. Consequently, RBM23 activated the NF-*κ*B signaling pathway and promoted expression of the proangiogenic cytokines selectively. *Results and Conclusion*. In summary, this study revealed that RBM23 promotes the angiogenesis properties of HCC via the NF-*κ*B signaling pathway. It may, therefore, be a potential therapeutic target for the treatment of hepatocellular carcinoma.

## 1. Introduction

Hepatocellular carcinoma (HCC) is the most common primary malignancy of liver cancer with a high global prevalence [1]. Owing to its high potential for vascular invasion, metastasis, and postoperative recurrence, most HCC patients die from a locally advanced or metastatic disease in a relatively short time [2]. However, the mechanisms responsible for HCC progression and metastasis are not clear at the moment [3]. Therefore, it is pending for us to identify new driving factors to deepen our understanding of the occurrence and development of this disease.

Angiogenesis, as we all know, is a hallmark of various malignancies, which is usually induced early during the multistage development of invasive cancers when we studied both in animal models and in humans [4]. HCC is a hypervascular malignancy accompanied by a high level of neovascularization, which ensures sufficient nutrition for the tumor and makes HCC cells prone to early metastasis through the blood tract [5]. The early blood tract metastasis not only makes a large number of patients lose the chance of radical surgery but also leads to a poor prognosis [6]. So far, only sorafenib has been approved by FDA for clinical treatment [7], but its effect on patients with advanced hepatocellular carcinoma is limited, and drug resistance is very easy to develop and accompanied by the emergence of some related complications [8], which brings great troubles for clinical use of liver cancer patients. This urged us to investigate further to discover new driving factors of HCC angiogenesis [9]. Based on this, we launched relevant research and initially identified RBM23 as a factor that promotes microvascular proliferation in hepatocellular carcinoma.

Patients with HCC often develop from chronic hepatitis and chronic cirrhosis [10]. During this development, a large number of inflammatory factors make the NF-*κ*B signaling pathway abnormally activated [11]. As is known to all, nuclear factor *κ*B (NF-*κ*B) is an important and well-researched transcription factor which regulates genes associated with a variety of cellular functions, such as cell proliferation [12], survival [13], angiogenesis [14], and cancer metastasis [15]. The dominant cellular NF-*κ*B is a heterodimer of p50 and p65 subunits, which are normally sequestered in the cytoplasm of nonstimulated cells. The p65 subunits are normally phosphorylated, then translocated into the nucleus once cells are exposed to extracellular stimuli [16]. In various diseases, the NF-*κ*B signaling pathway often has abnormal activation, resulting in the transcription of a large number of cancer-promoting genes, including cytokines that promote angiogenesis [17]. Therefore, to improve the prognosis of cancer, the NF-*κ*B signaling pathway activation must be terminated at the appropriate time. Thus, discovery of the molecule involved in controlling the aberrant activation of the NF-*κ*B signaling pathway is a potential target for therapeutic strategies against cancer.

In this study, we identified RBM23, which has not been researched before, as a novel promoter of HCC angiogenesis. We first analyzed the level of RBM23 in fresh tissue samples collected, which is positively correlated with microvessel density (MVD) in HCC tissues (indicated by CD31-positive cells). The results were further verified in the tissue microarray of hepatocellular carcinoma. Then, we analyzed the content of RBM23 in several HCC cell lines and established stable cell lines based on it, and various molecular and cytological experiments were used to demonstrate the promotion of HCC angiogenesis by RBM23 both in in vitro and in vivo assays. Mechanically, RBM23 improved the activity of the NF-*κ*B signaling pathway to produce more proangiogenesis cytokines by increasing the expression of RelA/p65.

## 2. Materials and Methods

### 2.1. Patients and Tissue Specimens

This study was conducted on 32 paraffin-embedded hepatocellular carcinoma (HCC) samples and corresponding noncancerous tissues, which were purchased from Servicebio (Wuhan, China); this process was approved by the Ethics Committee of the Affiliated Hospital of Nantong University, and relevant experiments were carried out. Additionally, 12 HCC specimens and matched adjacent noncancerous tissues from the Affiliated Hospital of Nantong University were frozen and stored in liquid nitrogen until further use. (The samples are well preserved in liquid nitrogen. The patients are all newly diagnosed hepatocellular carcinoma patients and have not received any form of anticancer treatment before surgery.) Prior consent from patients was obtained, and all the experimental procedures were approved by the Institutional Research Ethics Committee.

### 2.2. Cell Culture

Human hepatocellular carcinoma cell lines (Huh-7, SK-HEP-1, SMMC-7721, and HepG2) were purchased from the National Infrastructure of Cell Line Resource. They were cultured and passaged according to standard protocols. Cells were cultured in a humidified atmosphere containing 5% CO_2_ at 37°C in a DMEM (Hyclone) or RPMI medium (Hyclone) containing 10% FBS (Gibco, Invitrogen).

### 2.3. Real-Time PCR

Total RNA was extracted from HCC cells with the TRIzol Reagent (Invitrogen). The quality and concentration of the total RNA were determined using the Nanodrop 2000 spectrophotometer (Thermo Fisher Scientific, USA). Approximately 1 *μ*g of RNA was reverse-transcribed with HiScript III-RT SuperMix for qPCR (+gDNA wiper) (Vazyme, Nanjing, China). Quantitative real-time PCR was carried out in triplicate with AceQ Universal SYBR qPCR Master Mix (Vazyme, Nanjing, China) on a StepOne Plus Real-Time PCR System (Applied Biosystems. Foster City, CA). mRNA of *β*-actin served as the housekeeping gene. The primers used are listed as follows:

RT-B-actin-F: TCCCTGGAGAAGAGCTACG, RT-B-actin-R: GTAGTTTCGTGGATGCCACA, RT-IL-6-F: TGAAAGCAGCAAAGAGGCACTGG, RT-IL-6-R: CAGGCAAGTCTCCTCATTGAATCC, RT-IL-8-F: CTGTGTGAAGGTGCAGTTTTGCC, RT-IL-8-R: CGCAGTGTGGTCCACTCTCAATC, RT-VEGF-F: CATCACCATGCAGATTATGCG, RT-VEGF-R: CTATCTTTCTTTGGTCTGCATTCAC, RT-RelA-F: ATGTGGAGATCATTGAGCAGC, and RT-RelA-R: CCTGGTCCTGTGTAGCCATT.

### 2.4. Plasmids and Small Interfering RNAs

Complementary DNA (cDNA) of RBM23 was synthesized and cloned into pcDNA3.1-Puro-C-3Flag: OV-RBM23-F: TCTAGAATGGCATCTGATGACTTTGACATAG and OV-RBM23-R: GGTACCCATGGTCTGGGGGGTAAAG.

Knockdown of endogenous proteins was performed using two short hairpin RNA (shRNA) oligonucleotides which were cloned into pLKO.1-TRC-mCherry-BSD or pLVshRNA-EGFP (2A) Puro vectors: sh-RBM23-1: TCAGATACAGGCCGCTCTAAA, sh-RBM23-2: CTCCACTTGCCACTGGTTATA, sh-p65-1: CGGATTGAGGAGAAACGTAAA, and sh-p65-2: CACCATCAACTATGATGAGTT.

### 2.5. Western Blotting Analysis

All the cells and fresh tissues were lysed in radioimmunoprecipitation (RIPA) buffer (50 mM Tris (pH 7.4), 150 mM NaCl, 1% Triton X-100, 1% sodium deoxycholate, and 0.1% SDS) supplemented with 1% proteinase inhibitor (Beyotime, Shanghai, China). The lysed cells and tissues were quantitatively analyzed using a BCA kit (Beyotime, Shanghai, China). An equal amount of protein from each sample was separated by 10% SDS-PAGE and transferred to the PVDF membrane. The membranes were incubated overnight at 4°C with different primary antibodies and then incubated with the corresponding secondary antibodies (Beyotime, Shanghai, China): anti-RBM23 (bs-21292R) was bought from Bioss; anti-STAT3 (#12640), anti-IKba (#4814), anti-phospho-IKK*α*/*β* (Ser176/180) (#2697), anti-phospho-IKba (Ser32/36) (#9426), anti-NF-*κ*B p65 (#8242), and anti-NF-*κ*B p65 (Ser536) (#3033) were obtained from Cell Signaling Technology; anti-CD31 (ab9498) was from Abcam; and anti-IKKB (15649-1-AP), anti-p50/105 (14220-1-AP), anti-Fos (66590-1-Ig), anti-JUN (66590-1-Ig), and anti-GAPDH (60004-1-Ig) antibodies were purchased from Proteintech.

### 2.6. Cell Proliferation Assay

Approximately 2000 cells were seeded in each well of 96-well culture plates. A cell counting kit-8 assay (Vazyme, Nanjing, China) was used to evaluate the viability of cells according to the manufacturer's instructions. After incubation at 37°C for 2 h, the absorbance of the colored solution was measured using a microplate reader at 450 nm. The cell growth curve was established based on the value measured at different time points.

### 2.7. Immunofluorescence

Here, cells were cultured on coverslips, rinsed in PBS, fixed for 15 min with 4% formaldehyde, and permeabilized with 0.25% Triton X-100 for 20 min at room temperature. For immunofluorescence analysis, we used the following antibodies: RBM23 (1 : 100, Bioss) and RelA/p65 (1 : 100, Santa Cruz). Cell nuclei were stained with Hoechst 33258 (Beyotime, Shanghai, China).

### 2.8. Enzyme-Linked Immunosorbent Assay (ELISA)

To measure the levels of human IL-8, 3 × 10^5^ cells of different groups were plated in 6-well plates, and the supernatants were collected after 48 hours. The concentrations of human IL-8 in the supernatants were determined using the ELISA kit (MultiSciences, Hangzhou, China) according to the manufacturer's instructions.

### 2.9. Tube Formation Assay

A 96-well plate was coated with 100 *μ*l Matrigel (354234, BD Biosciences) and kept at 37°C for 1 hour. A total of 2 × 10^4^ HUVECs suspended in 100 *μ*l conditioned medium were applied to the precoated 96-well plate. Micrographs were taken after incubation at 37°C for another 8 hours.

### 2.10. Wound Healing Assay

The wound healing experiment was performed by setting up HUVECs at 2 × 10^5^ cells per well in a 6-well plate and allowing them to grow to confluence. Wounds were created using a 200 *μ*l pipette tip. The cells were then washed with phosphate-buffered saline (PBS) and incubated in the growth supernatant from different cells. Images were taken 48 hours later.

### 2.11. Animal Studies

Male BALB/c athymic nude mice (4 weeks old) were housed under standard conditions and cared for according to the institutional guidelines for animal care, which were obtained from the laboratory animal center of Nantong University. All of the animal experiments were approved by the Institutional Animal Care and Use Committee of Nantong University. To establish the subcutaneous mouse model, 1 × 10^6^ cells in 100 *μ*l of DMEM were subcutaneously injected into the nude mice. Six weeks later, the subcutaneous tumors were removed to conduct immunohistochemistry.

### 2.12. In Vivo Matrigel Angiogenesis Assay

A mixture containing 400 *μ*l of Matrigel (354234, BD Biosciences) and corresponding cell supernatant was subcutaneously injected into the dorsal surface of mice. Two Matrigel plugs were implanted in each mouse. The Matrigel plugs were retrieved five days later.

### 2.13. Immunohistochemistry (IHC)

Firstly, paraffin-embedded sections were incubated in 5% acetic acid and 0.2% trypsin solution at 37°C for 15 min for antigen retrieval. Then, sections were blocked in 10% (*v*/*v*) normal goat serum for 1 h at room temperature. Primary antibodies were diluted in 5% (*v*/*v*) normal goat serum and incubated overnight at 4°C in a humidified chamber. Biotin-labeled secondary antibodies were then used. Finally, visualization was achieved by using 3,3-diaminobenzidine (DAB).

### 2.14. Statistical Analysis

Data were analyzed using GraphPad Prism 7.0. Mean values of different groups were compared using unpaired Student's *t*-tests and ANOVA. *p* ≤ 0.05 was considered statistically significant. ^∗^*p* ≤ 0.05, ^∗∗^*p* ≤ 0.01, and ^∗∗∗^*p* ≤ 0.001.

## 3. Result

### 3.1. Expression Levels of RBM23 Are Positively Correlated with MVD in HCC Tissues

**Figure 1:**
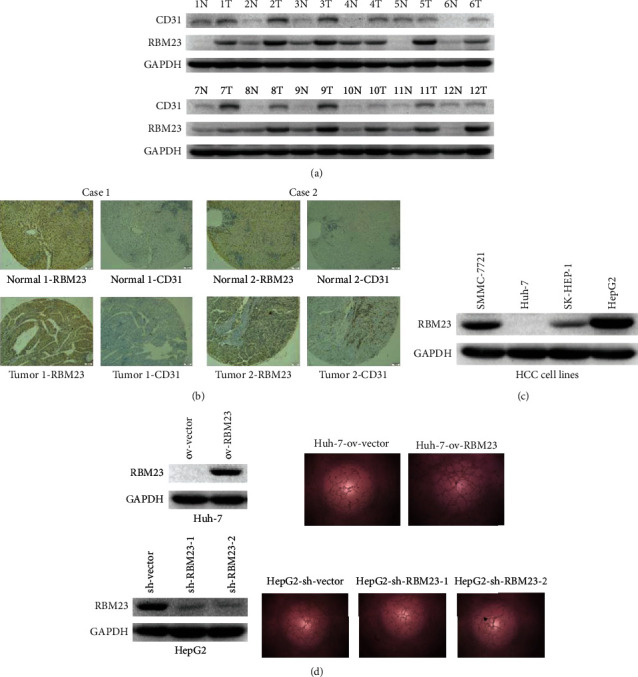
Expression levels of RBM23 positively correlated with MVD in HCC tissues. (a) Western blot analysis of the expression of RBM23 and CD31 in paired tumor tissues and matched normal tissues; GAPDH was used as a loading control. (b) IHC staining indicating higher RBM23 protein expression in HCC tissues than in normal liver tissues. The expression showed a high positive correlation with MVD (as indicated by CD31-positive cells) (1 cm represents 100 *μ*m). (c) Differences in RBM23 expression between different tumor cells. (d) The western blotting assays showing overexpression and knockdown of RBM23 in Huh-7 cells and HepG2 cells, respectively (left); RBM23 overexpression in Huh-7 cells promoted HUVEC tube formation whereas RBM23 knockdown in HepG2 cells inhibited HUVEC tube formation (right).

In order to investigate the role of RBM23 in hepatocellular carcinoma progression, we examined its expression in hepatocellular carcinomas and then compared with the adjacent normal tissues by western blotting assays. Results showed that the protein levels of RBM23 were significantly higher in most tumor samples when compared with the paired normal tissues (Figure 1(a) and Figure S1A). This suggested that RBM23 might be involved in the development of liver cancer. To investigate the correlation between RBM23 levels and MVD in HCCs, we detected the expression levels of RBM23 and CD31 in tumor specimens from 32 HCC patients by IHC staining in another queue. We observed a strong positive correlation between the levels of RBM23 and MVD (Figure 1(b)), which was also verified by western blotting assays in the previous queue (Figure 1(a)), suggesting that RBM23 might drive the development of hepatocellular carcinoma by affecting tumor angiogenesis. Furthermore, the protein levels of RBM23 were detected by western blot in different HCC cell lines (Figure 1(c)). Corresponding stable cell lines were established based on these results; based on the results above, we investigated the effect of tumor cells on the tube formation of HUVECs *in vitro*, which is crucial for tumor angiogenesis. The complete tubular structures formed by HUVECs were significantly increased and decreased in conditioned medium from Huh-7-ov-RBM23 and HepG2-sh-RBM23 cells, respectively, than in the corresponding control cells (Figure 1(d) and Figure S1B).

### 3.2. RBM23 Promotes Tumor Angiogenesis In Vitro

**Figure 2:**
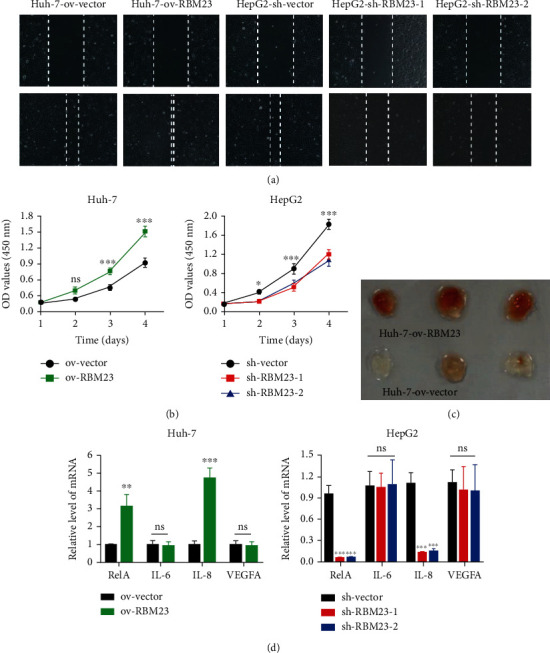
RBM23 promotes tumor angiogenesis in vitro. (a) Wound healing assay showing that supernatant from the Huh-7-ov-RBM23 cells highly promoted the migration of HUVECs *in vitro* compared to the corresponding cells. The stimulating effect of the medium from HepG2/-sh-RBM23 on the migration of HUVECs was not detectable. (b) Cell counting kit-8 assays showing RBM23 knockdown in HepG2 cells inhibited HUVEC growth whereas RBM23 overexpression in Huh-7 cells promoted HUVEC growth. (c) Images of Matrigel plugs retrieved, and there are significantly more blood vessels in plugs mixed with the cell supernatant of the overexpression group. (d) The mRNA levels of RelA, IL-6, IL-8, and VEGF from different cells as determined by real-time PCR analysis.

Through the above experiments, we found that there is a large clinical correlation between RBM23 and microvessel density (MVD), which can affect the complete tubular structures formed by HUVECs. Then, the functional role of RBM23 in angiogenesis was also determined through the investigation of *in vitro* HUVEC growth and migration by cell counting kit-8 assays and wound healing assays. As it was showed, the migration of HUVECs was inhibited in conditioned medium from HepG2-sh-RBM23 cells and promoted in the conditioned medium from Huh-7-ov-RBM23 cells compared with the corresponding controls (Figure 2(a)). Similar results were found in the cell counting kit-8 assays (Figure 2(b)). Moreover, to reveal the effect of conditioned medium from Huh-7-ov-RBM23 and corresponding control cells on angiogenesis in vivo, we mixed the cell supernatants with Matrigel and injected the mixture into a subcutaneous location and took out the plugs 5 days later (Figure 2(c)). On examining the mRNA level of IL-6, IL-8, and VEGF, which are the main cytokine that promotes angiogenesis, we found that RBM23 mainly affected IL8 and had a relatively small effect on IL-6 and VEGF (Figure 2(d)).

### 3.3. RBM23 Activates NF-*κ*B Activity and Promotes the Expression of Its Target Proangiogenesis Genes

**Figure 3:**
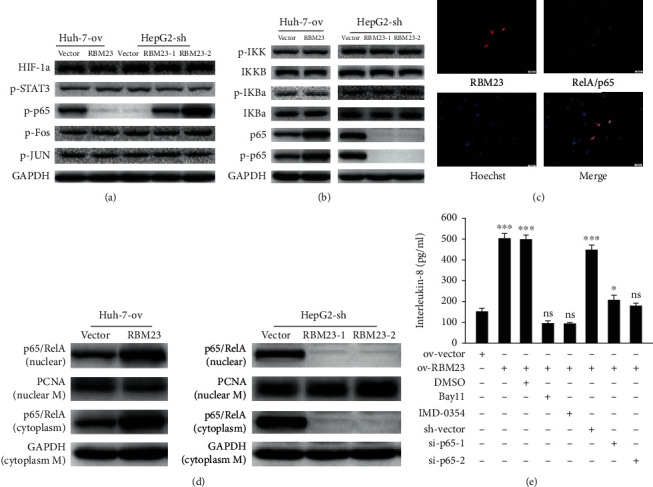
RBM23 activates NF-*κ*B activity and promot05es the expression of its target proangiogenesis genes. (a) Alteration of RBM23 did not affect the expression of the key molecules of related signaling pathways. (b) RBM23 positively regulated RelA/p65 without influencing the expression of other molecules in the NF-kappaB signaling pathway. (c) Immunofluorescence results showed that overexpression of RBM23 can increase the expression of RelA/p65 in Huh-7 cells (1 cm represents 100 *μ*m). (d) Western blot analysis of nuclear p65 and cytoplasmic p65 in different cells. (e) ELISA analysis of the IL-8 content from different supernatant of cells with different treatments.

To explore the specific mechanism by which RBM23 affects angiogenesis, we first examined the key molecules of related signaling pathways, including HIF-1a, NF-kappaB, STAT3, and AP1 pathways (the cells of each group were cultured in a hypoxic environment with 1% oxygen for 16 hours, and then, the expression of HIF-1a was detected). Western blotting assays revealed that RBM23 mainly activated the NF-*κ*B signaling pathway selectively (Figure 3(a)). Then, we investigated the potential mechanisms of the NF-*κ*B signaling pathway controlled by RBM23. No significant change in the expression of other important molecules in this signaling pathway was observed between the modified RBM23 expression and respective control cells. However, NF-*κ*B p65 and its phosphorylated (serine-536) levels decreased in HepG2-sh-RBM23 and increased in Huh-7-ov-RBM23 compared with the corresponding control cells (Figure 3(b)). Moreover, we verified that RBM23 could affect the NF-kappaB pathway using immunofluorescence (Figure 3(c)). Using cell fractionation and western blot, we found that both the cytoplasmic p65 and nuclear p65 were significantly increased upon RBM23 overexpression in Huh-7 cells and significantly decreased in RBM23 knockdown in HepG2 cells (Figure 3(d) and Figure S1C). These results indicated that RBM23 affected the activity of the NF-*κ*B signaling pathway by affecting the content of RelA/p65. The ELISA results also showed that the use of RelA/p65 short hairpin RNA or signaling pathway inhibitors (Bay11-7082, IMD-0354) could rescue the increase in IL-8 caused by overexpression of RBM23 in Huh-7 cells (Figure 3(e)). This further showed that RBM23 promoted tumor angiogenesis by activating the NF-*κ*B signaling pathway.

### 3.4. In Vivo RBM23 Promotes Angiogenesis in Cancer

**Figure 4:**
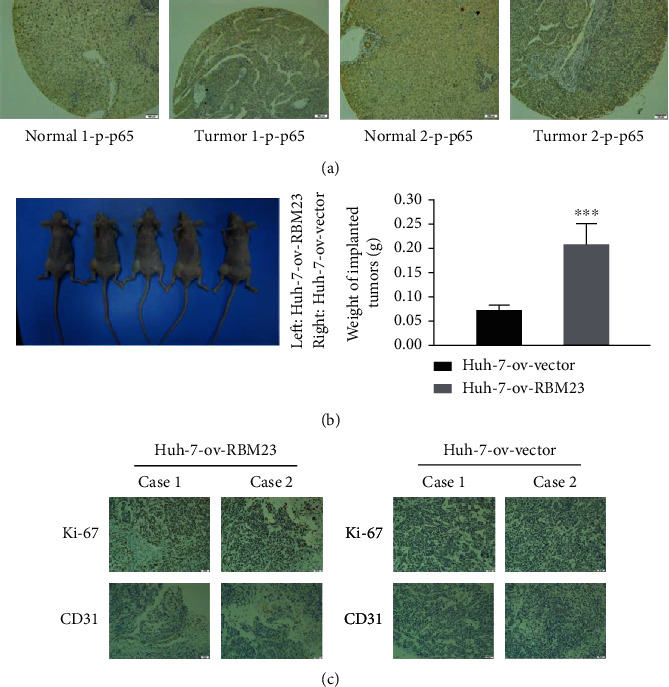
In vivo RBM23 promotes angiogenesis in cancer. (a) IHC staining indicating higher p-p65 protein expression in HCC tissues than in normal liver tissues. The expression showed a high positive correlation with RBM23 (1 cm represents 100 *μ*m). (b) RBM23 overexpression promoted the growth of tumor xenografts in nude mice. The left panel shows the photographs of the nude mouse. The right panel shows the weight of tumors after 28 days. (c) Immunohistochemistry (IHC) staining assays of the tumor microvessel density (indicated by CD31-positive cells) from subcutaneous tumors between different groups (1 cm represents 100 *μ*m).

The above experimental results showed that RBM23 can affect the NF-*κ*B signaling pathway, and then, we used the above tissue chip and select the same site to analyze the content of p-p65 (ser-536), which is found to be highly expressed in cancer tissues compared to the matched normal tissues and has a good correlation with RBM23 (Figure 4(a)). To investigate the role of RBM23 in tumorigenesis in vivo, Huh-7-ov-RBM23 and corresponding control cells were injected subcutaneously into nude mice, and the tumors were removed after six weeks (Figure 4(b)). The microvessel density was evaluated by CD31 immunohistochemical staining, and the tumor proliferation was evaluated by Ki-67 immunohistochemical staining. The results obtained proved that RBM23 could promote tumor neovascularization in vivo (Figure 4(c)).

## 4. Discussion

In this study, we found that RBM23 was highly expressed in hepatocellular carcinoma (HCC) and correlated with tumor microvessel density (as indicated by CD31-positive cells), suggesting that RBM23 may be involved in the formation of microvessels in HCC and promote the proliferation and distant metastasis of HCC.

Angiogenesis promotes rapid progress and early metastasis in hepatocellular carcinoma (HCC) [18]. This brings challenges to clinical treatment because a large number of patients lose the opportunity of surgery due to the early metastasis, which not only brings great challenges to the radical cure of hepatocellular carcinoma but also creates huge economic and social pressure for the society and individual patients [19]. In the course of clinical treatment, it can be found that a large number of patients have lost the chance of radical surgery due to the early metastasis of the intrahepatic portal vein system or the metastasis of distant organs through the bloodstream [20]. Nonetheless, it has also brought new ideas for treatment such as targeted therapy for tumor neovascularization. Sorafenib, a small inhibitor of several tyrosine protein kinases, contains vascular endothelial growth factor receptor, platelet-derived growth factor receptor, and Raf. Although sorafenib has been recently approved by the FDA for the treatment of advanced HCC [21] and showed improved survival rates in these patients, it still has huge risks and challenges [22]. At the same time, sorafenib has been the only first-line drug for advanced hepatocellular carcinoma for many years [23]. Therefore, it is urgent to identify new therapeutic targets to develop a treatment for HCC treatment. In this study, we identified RBM23 as a new molecule that promotes tumor angiogenesis, which provides a new perspective for us to deepen our understanding of angiogenesis in hepatocellular carcinoma.

Through further studies, we found that RBM23 promotes angiogenesis by promoting the expression and secretion of IL-8. From a mechanical point of view, RBM23 promotes IL-8 transcription by increasing the expression of RelA/p65 and then the activity of the NF-*κ*B signaling pathway.

The NF-*κ*B signaling pathway, a critically vital signaling pathway in tumorigenesis, regulates numerous downstream cancer-promoting genes, including some proangiogenesis cytokines, such as IL-6, IL-8, and VEGF [24]. Recently, researchers demonstrated that ubiquitination- and phosphorylation-mediated signaling transductions critically control the activation of the NF-*κ*B signaling pathway [25]; among them, IkBa-mediated cytoplasmic accumulation of the NF-*κ*B p65/p50 complex has been considered a key mechanism of terminating the NF-*κ*B signaling pathway [26], for the reason that deterring RelA/p65, the most important molecule in this signaling pathway, would completely block the activity of the NF-*κ*B signaling pathway [27]. For instance, Wang and colleagues reported in gastric cancer that CHIP interacted with RelA/p65 and promoted its ubiquitination and degradation via a proteasome terminating NF-*κ*B signaling pathway activity and inhibiting angiogenesis induced by IL-8 [28]. Besides, Liu et al. found in hepatocellular carcinoma that PROX1 elevated the level of RelA/p65 and stabilized RelA/p65 by recruiting ubiquitin-specific protease 7 (USP7). This prevented RelA/p65 from degradation thereby enhancing the activity of the signaling pathway [29].

However, it is not yet known whether there is a new regulatory mechanism for RelA/p65. In this study, RBM23 did not affect the activation and expression of IkBa in our study; unexpectedly, we found that RBM23 could improve the level of RelA/p65 mRNA, thereby increasing the activity of the NF-*κ*B signaling pathway to promote the angiogenesis of hepatocellular carcinoma.

In summary, this study demonstrates that RBM23 regulates the activity of the NF-*κ*B signaling pathway in HCC via directly targeting RelA/p65; these data revealed the RBM23-RelA/p65-NF-*κ*B axis as a mechanism of promoting the angiogenesis in hepatocellular carcinoma. At the same time, knocking down RBM23 inhibited HCC cell malignant phenotypes and, importantly, suppressed HCC angiogenesis by inhibiting NF-*κ*B activity through reducing the expression of RelA/p65 and downregulation of the proangiogenic cytokine IL-8. We propose that RBM23 may serve as a promising prognostic marker for hepatocellular carcinoma, and knocking down CHIP may be a novel strategy for antiangiogenesis therapy for human HCC.

## 5. Conclusions

In this study, we identified RBM23, a molecule that was not researched before, as a novel proangiogenic factor in hepatocellular carcinoma (HCC), which facilitates the angiogenesis of hepatocellular carcinoma (HCC) via modulating the NF-*κ*B signaling pathway, and through analysis of the clinical samples of multiple cohorts and performing in vivo experiments, we believe that RBM23 can be a new target for HCC treatments.

## Data Availability

The statistics data used to support the findings of this study are included within the supplementary information file.

## Supplementary Materials

Supplementary Materials The supplementary material is the quantitative data statistics for related experiments in the main text.
