# Supplementary material for: RBM23 Drives Hepatocellular Carcinoma by Activating NF-κB Signaling Pathway
Source: Biomed Res Int. 2021 Mar 17;2021:6697476. doi: 10.1155/2021/6697476 (PMC7994101; doi:10.1155/2021/6697476)
Supplement: Supplementary Materials — The supplementary material is the quantitative data statistics for related experiments in the main text. [file 6697476.f1.pdf]

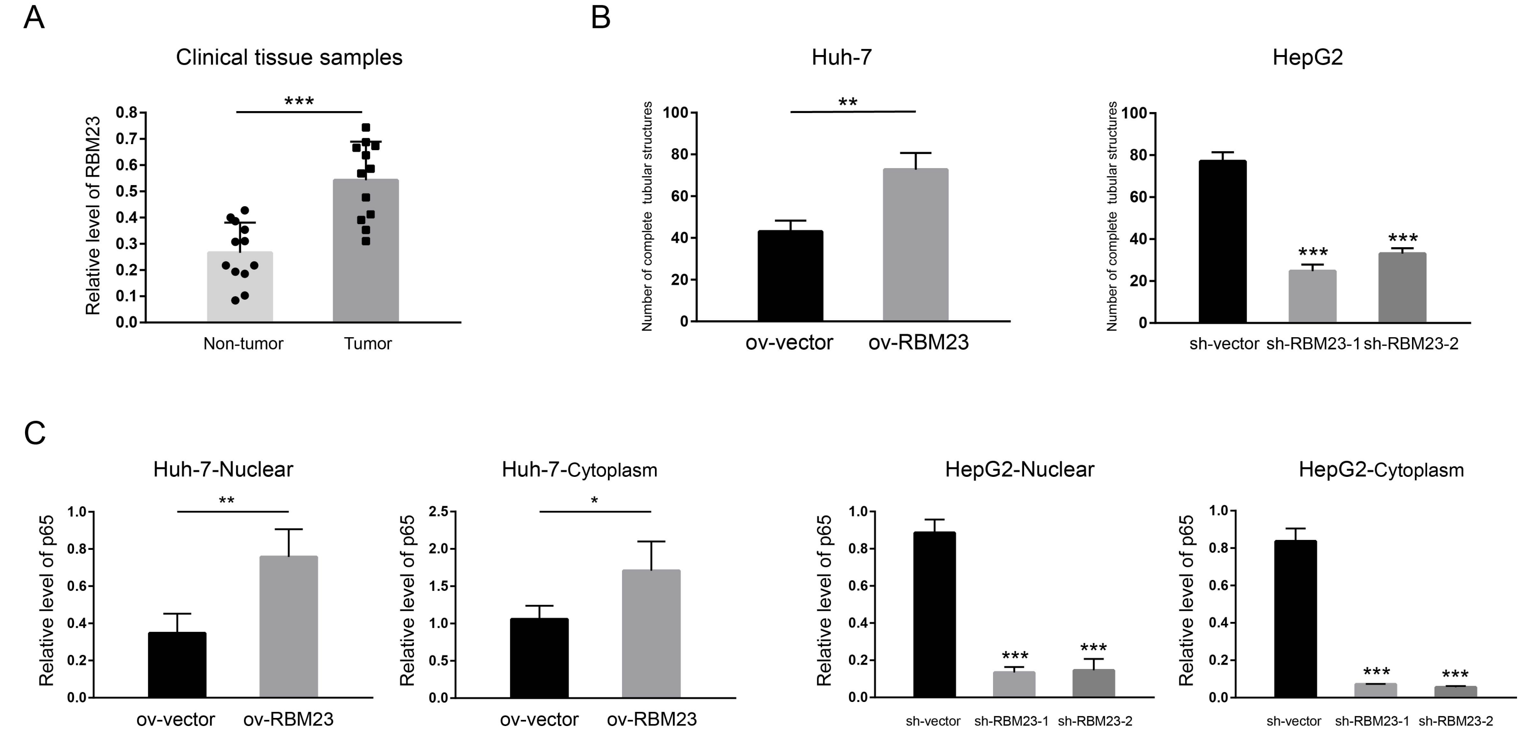

Figure S1. Statistical results of related experimental results

(A) Taking GAPDH as a reference, the expression of RBM23 in tumor tissues was significantly higher than that in corresponding adjacent tissues.

(B) Comparing the differences in the tube formation ability of human umbilical vein endothelial cells (HUVECs) under the stimulation of supernatant in different groups. Data from the average of three random field tube numbers.

(C) Compare the effect of RBM23 on RelA/p65 subcellular localization content in different experimental groups.
